# The IMAGEN study: a decade of imaging genetics in adolescents

**DOI:** 10.1038/s41380-020-0822-5

**Published:** 2020-06-29

**Authors:** Lea Mascarell Maričić, Henrik Walter, Annika Rosenthal, Stephan Ripke, Erin Burke Quinlan, Tobias Banaschewski, Gareth J. Barker, Arun L. W. Bokde, Uli Bromberg, Christian Büchel, Sylvane Desrivières, Herta Flor, Vincent Frouin, Hugh Garavan, Bernd Itterman, Jean-Luc Martinot, Marie-Laure Paillère Martinot, Frauke Nees, Dimitri Papadopoulos Orfanos, Tomáš Paus, Luise Poustka, Sarah Hohmann, Michael N. Smolka, Juliane H. Fröhner, Robert Whelan, Jakob Kaminski, Gunter Schumann, Andreas Heinz, IMAGEN consortium, Lisa Albrecht, Chris Andrew, Mercedes Arroyo, Eric Artiges, Semiha Aydin, Christine Bach, Tobias Banaschewski, Alexis Barbot, Gareth Barker, Nathalie Boddaert, Arun Bokde, Zuleima Bricaud, Uli Bromberg, Ruediger Bruehl, Christian Büchel, Arnaud Cachia, Anna Cattrell, Patricia Conrod, Patrick Constant, Jeffrey Dalley, Benjamin Decideur, Sylvane Desrivieres, Tahmine Fadai, Herta Flor, Vincent Frouin, Jürgen Gallinat, Hugh Garavan, Fanny Gollier Briand, Penny Gowland, Bert Heinrichs, Andreas Heinz, Nadja Heym, Thomas Hübner, James Ireland, Bernd Ittermann, Tianye Jia, Mark Lathrop, Dirk Lanzerath, Claire Lawrence, Hervé Lemaitre, Katharina Lüdemann, Christine Macare, Catherine Mallik, Jean-François Mangin, Karl Mann, Jean-Luc Martinot, Eva Mennigen, Fabiana Mesquita de Carvahlo, Xavier Mignon, Ruben Miranda, Kathrin Müller, Frauke Nees, Charlotte Nymberg, Marie-Laure Paillere, Tomas Paus, Zdenka Pausova, Jean-Baptiste Poline, Luise Poustka, Michael Rapp, Gabriel Robert, Jan Reuter, Marcella Rietschel, Stephan Ripke, Trevor Robbins, Sarah Rodehacke, John Rogers, Alexander Romanowski, Barbara Ruggeri, Christine Schmäl, Dirk Schmidt, Sophia Schneider, MarkGunter Schumann, Florian Schubert, Yannick Schwartz, Michael Smolka, Wolfgang Sommer, Rainer Spanagel, Claudia Speiser, Tade Spranger, Alicia Stedman, Sabina Steiner, Dai Stephens, Nicole Strache, Andreas Ströhle, Maren Struve, Naresh Subramaniam, Lauren Topper, Henrik Walter, Robert Whelan, Steve Williams, Juliana Yacubian, Monica Zilbovicius, C. Peng Wong, Steven Lubbe, Lourdes Martinez-Medina, Alinda Fernandes, Amir Tahmasebi

**Affiliations:** 1 Department of Psychiatry and Psychotherapy, Charité—Universitätsmedizin Berlin, Campus Charité Mitte, Berlin, Germany; 2 Department of Social Genetic & Developmental Psychiatry, Institute of Psychiatry, King’s College London, London, UK; 3 Department of Child and Adolescent Psychiatry and Psychotherapy, Central Institute of Mental Health, Medical Faculty Mannheim, Heidelberg University, Square J5, 68159 Mannheim, Germany; 4 Department of Neuroimaging, Institute of Psychiatry, Psychology & Neuroscience, King’s College London, London, UK; 5 Discipline of Psychiatry, School of Medicine and Trinity College Institute of Neuroscience, Trinity College Dublin, Dublin, Ireland; 6 University Medical Centre Hamburg-Eppendorf, House W34, 3.OG, Martinistr. 52, 20246 Hamburg, Germany; 7 Department of Cognitive and Clinical Neuroscience, Central Institute of Mental Health, Medical Faculty Mannheim, Heidelberg University, Square J5, Mannheim, Germany; 8 Department of Psychology, School of Social Sciences, University of Mannheim, 68131 Mannheim, Germany; 9 NeuroSpin, CEA, Université Paris-Saclay, F-91191 Gif-sur-Yvette, France; 10 Departments of Psychiatry and Psychology, University of Vermont, Burlington, VT 05405 USA; 11 Physikalisch-Technische Bundesanstalt (PTB), Abbestr. 2–12, Berlin, Germany; 12 Institut National de la Santé et de la Recherche Médicale, INSERM Unit 1000 “Neuroimaging& Psychiatry”, University Paris Sud, University Paris Descartes—Sorbonne Paris Cité, and Maison de Solenn, Paris, France; 13 Institut National de la Santé et de la Recherche Médicale, INSERM Unit 1000 “Neuroimaging & Psychiatry”, University Paris Sud, University Paris Descartes, Sorbonne Université, and AP-HP, Department of Child and Adolescent Psychiatry, Pitié-Salpêtrière Hospital, Paris, France; 14 Rotman Research Institute, Baycrest and Departments of Psychology and Psychiatry, University of Toronto, Toronto, ON M6A 2E1 Canada; 15 Department of Child and Adolescent Psychiatry and Psychotherapy, University Medical Centre Göttingen, von-Siebold-Str. 5, 37075 Göttingen, Germany; 16 Department of Psychiatry and Neuroimaging Center, TechnischeUniversität Dresden, Dresden, Germany; 17 School of Psychology and Global Brain Health Institute, Trinity College Dublin, Dublin, Ireland; 18 Berlin Institute of Health, Berlin, Germany; 19 Behavioural and Clinical Neuroscience Institute, Cambridge Univesity, Cambridge, UK; 20 Commissariat àl’Energie Atomique, Paris, France; 21 PERTIMM, Paris, France; 22 University of Nothingem, Nothingem, UK; 23 Deutsches Referenzzentrum für Ethik, Bonn, Deutchland Germany; 24 Delosis, London, UK; 25 Centre Nationel de Genotypage, Paris, France; 26 GABO:milliarium mbH & Co. KG, Munich, Germany; 27 School of Psychiology, University of Sussex, Brighton, UK

**Keywords:** Neuroscience, Psychology, Psychology, Neuroscience

## Abstract

Imaging genetics offers the possibility of detecting associations between genotype and brain structure as well as function, with effect sizes potentially exceeding correlations between genotype and behavior. However, study results are often limited due to small sample sizes and methodological differences, thus reducing the reliability of findings. The IMAGEN cohort with 2000 young adolescents assessed from the age of 14 onwards tries to eliminate some of these limitations by offering a longitudinal approach and sufficient sample size for analyzing gene-environment interactions on brain structure and function. Here, we give a systematic review of IMAGEN publications since the start of the consortium. We then focus on the specific phenotype ‘drug use’ to illustrate the potential of the IMAGEN approach. We describe findings with respect to frontocortical, limbic and striatal brain volume, functional activation elicited by reward anticipation, behavioral inhibition, and affective faces, and their respective associations with drug intake. In addition to describing its strengths, we also discuss limitations of the IMAGEN study. Because of the longitudinal design and related attrition, analyses are underpowered for (epi-) genome-wide approaches due to the limited sample size. Estimating the generalizability of results requires replications in independent samples. However, such densely phenotyped longitudinal studies are still rare and alternative internal cross-validation methods (e.g., leave-one out, split-half) are also warranted. In conclusion, the IMAGEN cohort is a unique, very well characterized longitudinal sample, which helped to elucidate neurobiological mechanisms involved in complex behavior and offers the possibility to further disentangle genotype × phenotype interactions.

## Introduction

Two decades ago, several groups started to associate genetic variants with measures of brain structure and function rather than clinically diagnosed disease categories [1–4]. For example, it was assumed that variance in the genetic constitution of monoamine transporters should have a stronger impact on in vivo transporter availability and, consequently, brain function than on subjective mood states [1, 2]. Likewise, genetic variants associated with the dopamine D2 receptor were associated with in vivo receptor availability [3, 5]. Shortly thereafter, genotype effects on MRI-derived functional brain activation rather than protein expression were studied, with a focus on working memory-dependent brain activation [6]. A meta-analysis of effect sizes reflecting the association of genetic variance with brain versus behavioral data confirmed that the assessed genetic variants displayed stronger associations with brain function than with cognition or clinical symptoms [7, 8]. However, many early candidate gene study findings failed to replicate and meta-analyses showed that the observed associations between genotype and functional activation were smaller than originally assumed [9]. In fact, most early studies were not sufficiently powered to reliably produce large effect sizes. Moreover, a variety of disease-related as well as comorbid factors including smoking or stress hormone activation can interact with genotype effects on brain correlates [10, 11], requiring adequate sample sizes to address complex interactions.

To address the issue of statistical power and to confirm whether brain structures and functions are directly associated with genetic variance or are a secondary consequence of the disorder (e.g., due to pharmacological drug effects), prospective long-term studies are required, which assess young participants before the manifestation of symptoms or clinical disorders. For this purpose, the IMAGEN consortium was established in 2010. It includes 8 European centers; each of them recruited at least 250 healthy adolescents aged 14, who have been followed up at ages 16, 19, and 22 [12].

Another important benefit of the shift towards large multidisciplinary collaborations such as IMAGEN and ABCD [13] is that it allows for data driven discovery science and out-of-sample prediction for in vivo imaging genetics researchers. The initial IMAGEN sample size (>2000) was large enough to assess the effect of previously identified SNPs or polygenic risk scores on functional activation and their interaction with additional factors including environmental measures [14–16] and comorbid factors such as smoking or stress hormone activation [10, 11]. Although not large enough for candidate gene or polygenic score construction [17], a phenotypically rich longitudinal dataset like IMAGEN is uniquely placed to identify how genes and behavior relate via psychological or neurobiological intermediate phenotypes.

Since its inception, the IMAGEN consortium has published a number of significant papers investigating how genetic and imaging findings contribute to specific traits, behaviors, symptoms, and disorders, for example with respect to impulsive decision making and drug consumption [18, 19]. Despite the 100+ IMAGEN publications in the last decade, no systematic review of IMAGEN findings has been published to date. Here we present all original IMAGEN imaging genetics papers, i.e., papers assessing effects of genetic variation on brain structure or functional brain activation during either reward anticipation, behavioral inhibition or processing of affective faces, and discuss their respective behavioral correlates with a focus on drugs of abuse including alcohol, tobacco, and cannabis.

## Methods

### Data sources

A systematic search for all IMAGEN publications was carried out by LMM and AR between October 1st, 2016, and December 31st, 2018. Relevant studies were identified using the list of published papers provided online by the IMAGEN consortium (https://imagen-europe.com/publications/) and by systematically searching the PUBMED database using the search terms “IMAGEN” and “consortium.” Study references of identified articles were additionally reviewed and taken into account. Altogether, we identified a total number of 110 papers from the IMAGEN consortium including manuscripts with IMAGEN as a contributor. Among these, 62 publications report interactions between genotype and functional or structural brain data.

### Study selection

**Fig. 1:**
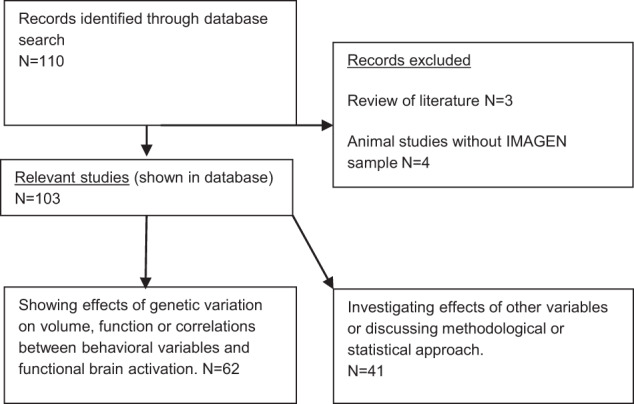
Selection process. Flow-chart of selection process of identified publications.

To provide a systematic account of all papers published by the IMAGEN consortium, we reviewed all identified papers. Abstracts were screened for relevance, and all identified articles were discussed by LMM, HW, AR, and AH. We excluded studies that were systematic literature reviews and animal studies that were not using the IMAGEN sample of participants (Fig. 1).

We categorized papers from the IMAGEN consortium with respect to whether genetic variations were associated with (1) structural brain measures or (2) with one of the three tasks applied for functional imaging, i.e., the stop signal task (SST), the monetary incentive delay task (MID) and the Emotional faces (EF), or (3) intake of drugs of abuse. The variety of genetic variants addressed in IMAGEN studies was classified into four categories with respect to the main function of putatively associated genes: (1) neurodevelopment, (2) apoptosis and cell cycle, (3) neurotransmission, and (4) metabolic or endocrine function [20, 21].

To facilitate further use of the papers by the scientific community, we created three tables with key findings of all imaging genetics papers published by the IMAGEN consortium. The tables provide a brief overview of every paper, listing the title of the paper, authors, journal, year, the single nucleotide polymorphisms (SNPs) investigated and the key imaging variable (brain volume, structure, or functional task) addressed by the study. Genotype effects are discussed with respect to findings of other papers by the IMAGEN consortium and by independent groups.

From the 110 papers, 7 were reviews that did not contain original data or focused exclusively on animal data; these 7 papers are not further discussed in this review. The remaining 103 studies reviewed here include 41 papers that focused on methodological issues or behavioral features, while 62 manuscripts reported genotype associations with brain structure or function or correlations between behavioral variables and functional brain activation.

## Results

### Description of identified studies

We identified 103 relevant original papers including published abstracts. Among these papers, 12 manuscripts reported associations between genetic variations and brain volume [4, 14, 19, 22–30] (Table 1). Furthermore, 16 manuscripts reported associations of genetic variation on functional brain activation elicited by the MID task [15, 31–45]; 6 papers described functional brain activation elicited by the SST [16, 18, 46–49], and 4 papers showed functional brain activation elicited by the EF [50–53] (Table 2). We identified 24 papers that did not address imaging genetics but reported correlations between behavioral variables and functional brain activation elicited by (1) the MID task (*n* = 13) [54–66], (2) the SST (*n* = 3) [67–69], and (3) the FRT (*n* = 8) [70–77] (Table 3).

**Table 1:** Influence of gene on brain structure volumes.

Gene SNP minor allele frequency	Title	Authors	Journal plus year	Number of participants	Mean age (years)	Main findings
Hippocampal findings						
Apo E Apolipoprotein E *rs429358* T/C (0.1506^a^) *rs7412* C/T (0.0751^a^) *E4 rs7412* C, (*rs429358* C) *E3 rs7412* T, *rs429358* C *E2* (*rs7412* T, *rs429358* T)	A multi-cohort study of ApoE 4 and amyloid- effects on the hippocampus in Alzheimer’s disease	Khan et al.	Journal of Alzheimer’s Disease, 2017	IMAGEN sample (*N* = 1387) Alzheimer’s disease (AD) and normal aging sample (*N* = 1781)	IMAGEN e2 carriers(14.4, SD 0.4) e3 and e4 carriers (14.5, SD 0.4)	AD and normal aging but not IMAGEN sample showed linear reduction in hippocampal volumes, with e4 carriers possessing the smallest volumes, e3 carriers possessing intermediate volumes, and e2 carriers possessing the largest volumes
Apo E Apolipoprotein E *rs429358* T/C (0.1506^a^) *rs7412* C/T (0.0751^a^) *E4 rs7412* C, (*rs429358* C) *E2* (*rs7412* T, *rs429358* T)	No differences in hippocampal volume between carriers and noncarriers of the ApoE E4 and E2 alleles in young healthy adolescents	Khan et al.	Journal of Alzheimer Disease, 2014	All participants *N* = 1412	APO E4, E2 noncarriers (14.45, SD 0.41) APOE4 carriers (14.44, SD 0.40) APO E2 carriers (14.45 ± 0.41)	No hippocampal volume or asymmetry differences between carriers and noncarriers of the ApoE E4 or E2 alleles, no dose-dependent effects of either allele
NR2F6 Nuclear receptor subfamily 2 group F member 6 *rs4808611* C/T (0.102^a^) USHBP1 Usher syndrome type-1C protein-binding protein 1 *rs35686037* C/T (0.088^a^) *rs12982178* T/C (0.103^a^) BABAM1 BRISC and BRCA1 A Complex Member 1 *rs10424178* C/T (0.173^a^) *rs10406920* C/T (0.112^a^) *rs8170* G/A (0.112^a^) TRPM8 Transient receptor potential cation channel *rs11901004* G/T (0.113) *rs17866592* C/T (0.135^a^) KIF26B Kinesin family member 26Brs*9919234* G/T (0.404^a^)	A genome-wide association study suggests novel loci associated with a Schizophrenia-related brain-based phenotype	Hass et al.	PLoS ONE, 2013	Consortia: MCIC^b^ (*N* = 241) ENIGMA^c^ (*N* = 7795) IMAGEN (*N* = 1663)	IMAGEN (~14, SD not reported) All other (~18, SD not reported)	Six SNPs on chromosome 19, located within or in close proximity to the genes NR2F6, USHBP1, and BABAM1, as well as four SNPs in three other genomic regions (chromosome 1, 2 and 10) correlated with hippocampal volume with significant p-values between 6.75×10 − 6 and 8.3×10 − 7. Allelic differences in rs4808611 and rs8170 strongly associated with differential mRNA expression in the cis-acting region. Various top-ranking SNPs of MCIC association analysis hits replicated in IMAGEN sample, two *KIF26B*SNPs; three *TRPM8* SNPs; 12 BABAM SNPS (e.g., rs2278897 with *p* = 5.1 × 10^−4^)
Located between HRK Harakiri, BCL2 Interacting Protein and FBXW8 F-Box And WD Repeat domain containing 8 *rs7294919* C/T (0.2119^a^) [influences expression of tescalcin gene] HMGA2 High mobility group AT-Hook 2 *rs10784502*C/T (0.3411^a^) DDR2 Discoidin domain receptor tyrosine kinase 2 *rs10494373*A/C (0.051^a^)	Identification of common variants associated with human hippocampal and intracranial volumes	Stein et al.	Nature Genetics, 2012	Total (*N* = 7795) IMAGEN (*N* = 518)	Total (39,9, SD9.24) IMAGEN (14.5, SD 0.4)	rs7294919 associated with hippocampal volume (*p* = 6.70 × 10^−16^). C allele of rs10784502 associated with intracranial volume and general intelligence by approximately 1.29 IQ points per risk allele (*P* = 1.12 × 10 − 12).
Putamen findings						

DAT1/SLC6A3 Dopamine transporter gene rs40184 C/T (0.477^a^)	Interaction between striatal volume and DAT1 polymorphism predicts working memory development during adolescence	Nemmi et al.	Developmental Cognitive Neuroscience, 2018	All participants (*N* = 487)	Baseline (~14, SD not reported) Follow-up (~19, SD not reported)	TC heterozygotes with a larger putamen at age 14 showed greater WM improvement at age 19 (*p* = 0.0009)
KTN1 Kinectin 1 *rs945270* G/C (0.343^a^) DCC DCC Netrin 1 Receptor *rs62097986* C/A (0.156^a^) BCL2L1 BCL2-Like 1 *rs6087771* T/C (0.299^a^) DLG2 Disks large homolog 2 *rs683250* A/G (0.417^a^) HRK Harakiri, BCL2 interacting protein *rs77956314* T/C (0.05^a^) MSRB3 Methionine sulfoxide reductase B3 *rs61921502* T/G (0.047^a^) FAT3 FAT Atypical Cadherin 3 *rs1318862* T/C (0.462^a^) CRHR1 Corticotropin releasing hormone receptor 1 *rs17689882* G/A (0.086^a^) HMGA2 High Mobility Group AT-Hook 2 *rs10784502* T/C (0.341^a^)	Common genetic variants influence human subcortical brain structures	Hibar et al.	Nature, 2015	Total: (*N* = 30,717) IMAGEN: (*N* = 1765)	IMAGEN: (14.6, SD 0.4)	Five genetic variants were associated with the volumes of the putamen and caudate nucleus. (rs945279, rs62097986, rs6087771, rs683250, rs1318862). Strongest effects for putamen was in rs945270 influencing the expression of the KTN1 gene (*p* = 1.08 × 10^-33^). Three loci replicated with previously established association with hippocampal and intracranial volume (rs77956314, rs6192150, rs 17689882) Rs10784502 did not survive genome-wide significance considering association with intracranial volume.

Cortex finding						
EFhd2 EF-Hand Domain Family Member D2 rs112146896 C/G (0.12^a^)	EFhd2/Swiprosin-1 is a common genetic determinator for sensation-seeking/low anxiety and alcohol addiction	Mielenz et al.	Molecular Psychiatry, 2017	All participants (*N* = 1980)	All participants (14.4, SD 0.41)	Minor C allele of rs112146896 shows a positive significant association with lifetime alcohol intake (EF = 0.099), a nominally positive association with binge drinking (EF = 0.055) and a negative association with anxiety. Negative association between lifetime alcohol intake and superior frontal gyrus volume.
Polygenic risk sore ➢ 20.000 SNPs CNR1 Cannabinoid receptor 1	Early cannabis use, polygenic risk score for schizophrenia, and brain maturation in adolescence	French et al.	JAMA Psychiatry, 2015	Cohorts: SYS^d^ (*N* = 949) ALSPAC^e^ (*N* = 295) IMAGEN (*N* = 333)	All participants (16.4, SD 1.0) IMAGEN Baseline (14.5, SD 0.4) Follow-up (18.5, SD not reported)	Negative association was between cannabis use in early adolescence and cortical thickness in male participants with a high polygenic risk score. Male participants showed an interactive effect of the high risk score and cannabis use on decreased cortical thickness from 14.5 to 18.5 years of age
NPTN Neuroplastin *rs7171755* G/A (C) (0.474^a^)	Single nucleotide polymorphism in the neuroplastin locus associates with cortical thickness and intellectual ability in adolescents	Desrivières et al.	Molecular Psychiatry, 2013	All participants (*N* = 1583)	All participants (14.4, SD 0.7)	Variant of rs7171755 (risk = minor allele A) is associated with smaller cortical thickness in frontal and temporal lobes, affecting left hemisphere (*p* = 1.12 × 10^−7^) more than right hemisphere and decreased verbal and non-verbal IQ
Other structural findings						
APOE Apolipoprotein E *E4 rs7412* C, (*rs429358* C) *E3 (rs7412* T, *rs429358* C) *E2* (*rs7412* T, *rs429358* T)	Tract based spatial statistic reveals no differences in white matter microstructural organization between carriers and noncarriers of the APOE ɛ4 and ɛ2 Alleles in Young Healthy Adolescents	Dell’Acqua et al.	Journal of Alzheimer’s Disease, 2015	All participants (*N* = 575)	All participants(14.4, SD 0.5)	Microstructural properties of white matter are not associated with the APOE ɛ4 and ɛ2 alleles in adolescents
Set of 136 genes	Correlated gene expression supports synchronous activity in brain networks	Richiardi et al.	Science, 2015	All participants (*N* = 259)	All participants(~14, SD not reported)	A set of 136 genes was significantly enriched for ion channels and polymorphisms in this gene set significantly affect resting-state functional connectivity, mostly within but also between functional networks
KDM4C Lysine-specific demethylase 4C *rs10511260* T/G (0.177^a^) *rs10758821* G/T (0.465^a^) *rs10975990* A/G (0.424^a^) PKNOX2 PBX/Knotted 1 Homeobox 2 *rs10893366* C/T (0.285^a^) rs750338 A/G (0.358^a^); ADH1C Alcohol Dehydrogenase 1C*rs1789891* C/A (0.095^a^) ANKS1A Ankyrin Repeat And Sterile Alpha Motif Domain Containing 1A *rs2140418* C/T (0.432^a^) KIAA0040 Uncharacterized protein *rs6701037* A/C (0.287^a^) KCNMA1 Potassium Calcium-Activated Channel Subfamily M Alpha 1 *rs717207*G/T (0.124^a^) RASGRF2 Ras Protein Specific Guanine Nucleotide Releasing Factor 2 *rs26907* G/A (0) *rs2369955* A/C (0.157^a^) nearMARK1 Microtubule Affinity Regulating Kinase 1 *rs7530302* G/A (0.135^a^) PECR Peroxisomal Trans-2-Enoyl-CoA Reductase *rs7590720* A/G (0.284^a^) EFNA5 Ephrin A5 *rs770182* T/G (0.323^a^)	Neuropsychosocial profiles of current and future adolescent alcohol misusers	Whelan et al.	Nature, 2014	IMAGEN Controls (*N* = 150) Current binge drinkers (*N* = 115) Future binge drinkers (*N* = 121)	IMAGEN Controls (14.5, SD 0.4) Current binge drinkers (14.6, SD 0.4) Future binge drinkers (14.5, SD 0.4)	Geneticvariantsrs2140418T, rs2369955C, rs7530302A, rs7590720G,and rs7916403T associated with current binge drinking; rs10758821T, rs10893366T, rs2140418T, and rs2369955C were predictive of future binge drinking

^a^Global minor allele frequency based on 1000Genome phase 3 genotype data (1000 Genomes Project Consortium. (2015). A global reference for human genetic variation. Nature, 526(7571), 68–74.)

^b^*MCIC* The Mind Clinical Imaging Consortium.

^c^*ENIGMA* Enhancing Neuro Imaging Genetics through Meta Analysis.

^d^*SYS* Saguenay Youth Study.

^e^*ALSPAC* Avon Longitudinal Study of Parents and Children.

**Table 2:** Genetic effects on functional brain activation elicited by MID, SST and EF task.

SNP	Title	Authors	Journal plus year	Number of participants	Age	Main findings
MID						
DRD1 D1 dopamine receptor rs686, A/G (0.409^a^) PPP1R1B protein phosphatase 1 regulatory inhibitor subunit 1B *rs8769* G/A (0.06^a^) DRD2 D2 dopamine receptor *rs12364283* A/G (0.07^a^) ANKK1 Ankyrin repeat and kinase domain containing 1, *rs1800497* G/A (0.188^a^)	Modulation of orbitofrontal-striatal reward activity by dopaminergic functional polymorphisms contributes to a predisposition to alcohol misuse in early adolescence	Baker et al.	Psychological Medicine, 2018	All participants (*N* = 1840)	All participants (14.55, SD = 0.447)	Functional polymorphism rs686 of the D1 dopamine receptor (DRD1) (p = 0.01) gene and Taq1A of the ANKK1 gene (*p* = 0.002) influenced medial and lateral OFC activation during reward anticipation. rs686 of the DRD1gene was indirectly related to early onset of alcohol misuse through a medial orbitofrontal cortex × ventral striatum interaction.
TTC12 tetratricopeptide repeat domain 12 ANKK1 ankyrin repeat and kinase domain containing 1 DRD2 dopamine receptor 2 — 33 SNPs, e.g., rs2236709 A/G (0.2588^a^)	A neurobiological pathway to smoking in adolescence: TTC12-ANKK1-DRD2 variants and reward response	Macare et al.	European Neuropsychopharmacology, 2018	NFBC1966 (*N* = 4512), NFBC1986 (*N* = 4307) ALSPAC (*N* = 3674) IMAGEN (*N* = 1591)	NFBC1966 (14, SD not reported) NFBC1986 (16, SD not reported) ALSPAC (15, SD not reported) IMAGEN (14, SD not reported)	The minor G allele was linked to an increased ventral-striatal BOLD response during reward anticipation (*p* = 0.032) and with higher *DRD2* gene expression in the striatum (*p* = 0.013). Same Allele was associated with self-reported smoking and higher plasma cotinine levels
GABRB1 Gamma-aminobutyric acid receptor subunit Beta-1 *rs2044081* C/T (0.148^a^)	GABRB1 Single Nucleotide Polymorphism Associated with Altered Brain Responses (but not Performance) during Measures of Impulsivity and Reward Sensitivity in Human Adolescents	Duka et al.	Frontiers in Behavioral neuroscience, 2017	All participants (*N* = 1299)	All participants (14, SD not reported)	Allele was not associated with an impulsive or reward-sensitivity phenotype as measured by SST and MID-Diff performance. Increased BOLD response in the right hemisphere inferior frontal gyrus, left hemisphere caudate/insula and left hemisphere inferior temporal gyrus during MID performance was higher in the minor (T) allelic group (*p* < 0.005). In contrast, during SST performance, the BOLD response found in the right hemisphere supramarginal gyrus, right hemisphere lingual and left hemisphere inferior parietal gyrus indicated reduced responses in the minor genotype (*p* < 0.005)
KTN1 Kinectin 1 *rs945270* C/G (0.3429^a^)	Impact of a common genetic variation associated with putamen volume on neural mechanisms of attention deficit/hyperactivity disorder	Xu et al.	Journal of the American Academy of Child & Adolescent Psychiatry, 2017	All participants (*N* = 1129)	All participants (14.4, SD 0.4)	rs945270 C allele associated with lower ADHD symptoms. rs945270 C allele associated with higher putamen activation during reward anticipation in females and with lower putamen activation during successful response inhibition in males
OPRL1 Opioid related nociceptin receptor 1 11 SNPs e.g., *rs1335579* A/G (0.214^a^)	Methylation of OPRL1 mediates the effect of psychosocial stress on binge drinking in adolescents	Ruggeri et al.	Journal of Child Psychology and Psychiatry, 2017	All participants (*N* = 325)	All participants (14, SD not reported)	Methylation levels in intron 1 of OPRL1 are associated with higher psychosocial stress and higher frequency of binge drinking. In individuals with low methylation of OPRL1, frequency of binge drinking is associated with stronger BOLD response in the ventral striatum during reward anticipation.
OPRM1 Opioid receptor Mu 1 *rs1799971* A/G (0.223^a^) *rs563649* C/T (0.1084^a^)	Brain substrates of reward processing and the m-opioid receptor: a pathway into pain?	Nees et al.	Pain, 2017	All participants Baseline (*N* = 644) Follow-up (*N* = 44)	All participants Baseline (14.58, SD 0.39) Follow-up (16.73, SD 0.21)	Functional activation of the dorsal striatum during reward feedback predicted pain complaints independent of genetic variance. T allele of rs563649 had more pain complaints than CC-allele carriers. Relationship of pain complaints and activation in the periaqueductal gray and ventral striatum in carriers of theT-allele of rs563649
	Polygenic risk of psychosis and ventral-striatal activation during reward processing in healthy adolescents	Lancaster et al.	The American Journal of Psychiatry, 2016			
KALRN kalirin RhoGEF kinase *rs6438839*, G/A (0.17) *rs4634050* (C/A) (0.22)	Mouse and human genetic analyses associate kalirin with ventral striatal activation during impulsivity and with alcohol misuse	Peña-Oliver et al.	Frontiers in Genetics, 2016	All participants (*N* = 1423)	All participants (14.43, SD = 0.41)	G major allele of the SNP rs6438839 in the KALRN gene was significantly associated with increased ventral striatum activation during reward anticipation (*p* = 5.9 × 10^−3^). A minor allele of SNP rs4634050, belonging to the same haplotype block, was associated with increased frequency of binge drinking (*p* = 4.87 × 10^−2^).
VPS4A vacuolar protein sorting-associated protein 4A *rs16958736* C/T (0.111)	Neural basis of reward anticipation and its genetic determinants	Tianye et al.	Proceedings of the National Academy of Science USA, 2016	All participants (*N* = 1423)	All participants (14.44, SD = 0.42)	The major C allele was associated with decreased activation in the striatal node during reward anticipation, although it did not reach the commonly used threshold for genome-wide significance. Lower activation in striatal node was associated with premature responding (*p* = 5.0 × 10 − 6).
EHD4 EH-domain containing 4 *Haplotype blockconsisting ofrs 1648821* C/T(0.126^a^)	A translational systems biology approach in both animals and humans identifies a functionally related module of accumbal genes involved in the regulation of reward processing and binge drinking in males	Stacey et al.	Journal of Psychiatry and Neuroscience, 2016	All participants (*N* = 907)	All participants (14.42, SD 0.41)	Functional activation of the right but not left ventral striatum during reward anticipation was significantlyassociatedwithhaplotype block 3 consisting ofrs 1648821 and 5 other SNPs in EHD4. M5 module, a functional gene-clusterassociated with mesolimbic dopamine signaling was linked to binge-drinking in both mice and male human adolescents
BDNF Brain-derived neurotrophic factor *rs6265* C/T (0.201^a^)	BDNF Val66Met and reward-related brain function in adolescents: role for early alcohol consumption	Nees et al.	Alcohol, 2015	All participants (*N* = 530)	All participants Baseline (14.33, SD 0.98) Follow-up: (16.28, SD 0.88)	Val/Val homozygotes versus Met -carriers had lower functional activation in putamen during reward anticipation. Low putamen activation during reward feedback in Met-carriers was associated with alcohol consumption at follow-up (EF = 0.024). Functional putamen activation during reward feedback in Met- but not Val/Val carriers at baseline predicted level of alcohol consumption 2 years later (EF = 0.011)
RSU1 Ras suppressor protein 1 *rs7078011*C/T (0.041)	Rsu1 regulates ethanol consumption in *Drosophila* and humans	Ojelade et al.	Proceedings of the National Academy of Science USA, 2015	All participants (*N* = 1303)	All participants (14.4, SD 0.4)	Polymorphisms in RSU1 are associated with functional activation in the ventral striatum during reward anticipation alcohol consumption in adolescents
DRD2/ANKK1 Dopamine receptor D2/ Ankyrin repeat and kinase domain containing 1, *rs1800497* G/A (0.326^a^)	DRD2/ANKK1 polymorphism modulates the effect of ventral striatal activation on working memory performance	Nymberg et al.	Neuropsychopharmacology, 2014	All participants (*N* = 1080)	All participants (14.4, SD 0.4)	Higher ventral striatum and caudate activation during reward feedback significantly associated with higher WM performance (EF = 0.010). This effect was only significant in carriers of the minor A allele (EF = 0.07)
CHRNA5–CHRNA3–CHRNB4 Cholinergic receptor nicotinic alpha subunit (5–3–4) *rs578776* G/A (0.445^a^) *rs1051730* G/A (0.168^a^)	Genetic risk for nicotine dependence in the cholinergic system and activation of the brain reward system in healthy adolescents	Nees et al.	Neuropsychopharmacology, 2013	All participants (*N* = 999)	All participants (14.55, SD 0.25)	Carriers of the rs578776 GG allele versus A- carriers had significantly lower functional activation elicited by reward feedback in the right ventral and dorsal ACC(EF = 0.3157)
MAOA Monoamine oxidase A *rs12843268* A/G (0.470^a^)	Neural mechanisms of attention deficit/hyperactivity disorder symptoms are stratified by MAOA genotype	Nymberg et al.	Biological Psychiatry, 2013	All participants (*N* = 648)	All participants (14.4, SD 0 .4)	In male rs12843268 A hemizygotes, ADHD symptoms are associated with lower functional activation of the VS during reward anticipation and lower inferior frontal gyrus BOLD response during response inhibition (SST). In G hemizygotes, right inferior frontal gyrus activation during response inhibition 8ST) was positively correlated with ADHD symptoms in the presence of increased ventral striatal functional activation during reward anticipation (EF = 0.0053)
TNM4 Teneurin transmembrane protein 4 *rs12576775* A/G (0.104^a^)	The risk variant in ODZ4 for bipolar disorder impacts on amygdala activation during reward processing.	Heinrich et al.	Bipolar Disorders, 2013	All participants (*N* = 485)	All participants (14.26, SD 0.30)	rs12576775 G allele carriers had an increased functional activation in the amygdala during reward anticipation and feedback
Rasgrf2 Ras protein specific guanine nucleotide releasing factor 2 *rs26907* G/A (0.157^a^)	RASGRF2 regulates alcohol-induced reinforcement by influencing mesolimbic dopamine neuron activity and dopamine release.	Stacey et al.	PNAS, 2012	All participants (*N* = 612) (in abstract *n* = 663 mentioned)	All participants (14.44, SD 0.40)	Ethanol-induced dopamine release blunted in Rasgrf2 k.o.mice. A Rasgrf2 haplotype block containing rs26907, a SNP previously associated with alcohol intake, was significantly associated with ventral striatal functional activation during reward anticipation (EF = 0.0205)
SST						
PSD3 Pleckstrin and Sec7 domain containing 3 *rs13265422* G/A (0.472^a^)	The Arf6 activator Efa6/PSD3 confers regional specificity and modulates ethanol consumption in Drosophila and humans	Gonzalez et al.	Molecular Psychiatry, 2017	IMAGEN sample for association analyses (*N* = 1363) IMAGEN sample for fMRI task (*N* = 1771) SAGE sample (*N* = 2544)	IMAGEN sample for association analysis (16.46,SD 0.51) IMAGEN sample for fMRI task (14.43, SD 0.42) (fMRI task)	Haplotype containing rs13265422 and PSD3 Minor G allele of rs13265422 were significantly associated with increased frequency of alcohol consumption (EF = 0.2167) and binge drinking in the last 30 days (EF = 0.1125). Haplotype containing rs13265422 also modulates PFC activation during SST
PPM1G Protein phosphatase, Mg2 + /Mn2+ Dependent 1G *rs7602534* C/T (0.378^a^) *rs11675428* A/C (0.050^a^) *rs704791* T/C (0.454^a^) *rs1260342* G/T (0.454^a^) *rs2384629* G/A (0.0078^a^)	Association of protein phosphatase PPM1G with alcohol use disorder and brain activity during behavioral control in a genome-wide methylation analysis	Ruggeri et al.	American Journal of Psychiatry, 2015	IMAGEN (499 adolescents) 18 pair of twins	IMAGEN (~14, SD not reported) Twins first follow-up (~16, SD not reported)	Hypermethylation of PPM1G was positively associated with high daily alcohol intake drinking, impulsivity and functional activation of the right subthalamic nucleus during stop success in the SST task (η2 = 0.013). *PPM1G* genotype and methylation profile were not associated, thus indicating environmental causes
COMT Catecholamine-0-methyl-transferase *rs4680* G/A (0.369^a^)	Sex differences in COMT polymorphism effects on prefrontal inhibitory control in adolescence	White et al.	Neuropsychopharmacology, 2014	All participants (*N* = 1133)	All participants(14.45, SD 0.41)	Male but not female Val homozygotes displayed elevated functional activation in pre supplementary motor area (pre- SMA) during successful-inhibition trials and in both pre-SMA and inferior frontal cortex during failed-inhibition trials compared with other genotypes (EF = 0.2038)
AMBRA1 Autophagy/beclin-1 regulator *rs11819869* C/T (0.250^a^)	From gene to brain to behavior: schizophrenia-associated variation in AMBRA1 alters impulsivity-related traits	Heinrich et al.	European Journal of Neuroscience, 2013	All participants (*N* = 848)	All participants(14.44, SD 0.41)	T-risk allele carriers in the rs11819869 showed higher delay aversion (EF = 0.1634) and functional activation in an orbitofrontal target region during the SST(EF = 0.1651)
SLC6A2 Solute carrier family 6 member 2 *rs36024* G/A (0.463^a^)	Adolescent impulsivity phenotypes characterized by distinct brain networks	Whelan et al.	Nature Neuroscience, 2012	All participants (*N* = 1896)	All participants (14.55 ± 0.447)	Hypofunctioning of a specific orbitofrontal cortical network was associated with likelihood of initiating drug use in early adolescence. Right inferior frontal activity was related to the speed of the inhibition process and use of illegal substances and associated with genetic variation in a norepinephrine transporter gene
ADRA2B Adrenoceptor Alpha 2B *SNPs not reported*	A large-sample fMRI study (*n* = 1252) of individual differences in inhibitory control	Whelan et al.	Human brain mapping Quebec abstract book, 2011	All participants (*N* = 1252)	All participants (14.51, SD 0.86)	Participants with subclinical features of ADHD showed reduced functional activation during stop failure in bilateral putamen, pallidum,caudate,bilateral insula, ACC and IFG. ADRA2B was associated with activation during stop success in right lateralized IFG, insula and ACC
Emotional faces task						
GWAS with 463,940 SNPs	Genetic risk for schizophrenia and autism, social impairment and developmental pathways to psychosis	Velthorst et al.	Translational Psychiatry, 2018	All participants (*N* = 2096) Subgroup used for structural equation modeling (SEM) (*N* = 643)	All participants (14.46, SD 0.41) SEM (14.44, SD 0.43) 14.44 (0.43)	Reduced brain activity to emotional stimuli (*p* = 0.009) as well as social impairments in late adolescence (*p* < 0.001) and high polygenic risk for schizophrenia (*p* = 0.014) independently contributed to the severity of psychotic experiences at age 18
CB1R Cannabinoid receptor 1 *rs1049353* C/T (0.129^a^) *rs806377* T/C (0.490^a^)	The role of the cannabinoid receptor in adolescents′ processing of facial expressions	Ewald et al.	Cognitive Neuroscience, 2016	All participants (*N* = 583)	All participants (14.38, SD 0.96)	A-allele versus GG-carriers in rs1049353 displayed earlier recognition of facial expressions changing from anger to sadness (EF = 0.1735) or fear(EF = 0.175), increased functional activation elicited by angry but not neutral faces in the amygdala (EF = 0.1775) and insula (EF = 0.1981) No significant effects were observed for rs806377
OXTR Oxytocin receptor 23 SNPs, e.g., *rs237915* T/C (0.159^a^)	Oxytocin Receptor Genotype modulates ventral-striatal activity to social cues and response to stressful life events	Loth E et al.	Biological Psychiatry, 2014	All participants (*N* = 1445)	All participants (14.4, SD 0.7)	rs237915 CC-homozygoteshad significantly lower vs activation elicited by angry faces than T-allele carriers (left vs activity EF = 0.1779, right vs ES = 0.1672). In environments with low stressful life events, rs237915 CC homozygote girls had more emotional problems and boys had more peer problems. In high stressful environments, T-allele carriers had more clinical problems than CC homozygotes
GWAS with 511.089 SNPs	Global genetic variations predict brain response to faces	Dickie et al.	PLoS Genetics, 2014	All participants (*N* = 1620)	All participants (~14, SD not reported)	A significant proportion of the brain response to ambiguous but not angry facial expressions was predicted by common genetic variance in 9 out of 25 regions constituting a face network. The strength of the genotype-phenotype relationship varied according to the number of functional connections of each region, the identified 9 regions displayed the highest inter-individual variability in the number of connections with other network nodes

^a^Global minor allele frequency based on 1000Genome phase 3 genotype data (1000 Genomes Project Consortium. (2015). A global reference for human genetic variation. Nature, 526(7571), 68–74).

**Table 3:** Functional activation elicited by MID, SST, and EF task.

Title	Authors	Journal plus year	Number of participants	Age in years (mean)	fMRI task	Main finding
Examination of the neural basis of psychotic-like experiences in adolescence during reward processing	Papanastasiou et al.	Journal of American Psychiatry, 2018	All participants (*N* = 298)	Baseline (14.47, SD = 0.39) Follow-up (19.02, SD = 0.76)	Monetary incentive delay task (MID)	Between baseline and follow-up, brain activation in two regions within the left and right middle frontal gyri increasedduring reward anticipation (*p* = 0.02; *p* = 0.03, respectively); there was no main group effect between high vs low psychotic-like experiences.
Epigenetic variance in dopamine D2 receptor: a marker of IQ malleability?	Kaminski et al.	Translational Psychiatry, 2018	All participants (*N* = 1475)	All participants (14.43, SD = 0.45)		Functional striatal activation elicited by temporarily surprising reward-predicting cues (from MID task) as well as polygenic scores for intelligence and epigenetic modification of *DRD2* gene and gray matter density in striatum were associated with general IQ
Blunted ventral striatal responses to anticipated rewards foreshadow problematic drug use in novelty-seeking adolescents	Büchel et al.	Nature Communications, 2017	Healthy controls (*N* = 72) Problematic drug use (*N* = 72)	Healthy controls (14.48, SD 0.40) Problematic drug use (14.38, SD 0.45)		During reward anticipation, lower functional activation in dorsolateral PFC, ventral striatum and midbrain predict drug use at age 16
Ventral Striatum Connectivity During Reward Anticipation in Adolescent Smokers	Lee et al.	Developmental neuropsychology, 2016	All (*N* = 206)	All Participants (~ 14, SD not reported)		Increased smoking frequency was associated with increased connectivity between ventral striatum and regions involved in saliency and valuation, including the orbitofrontal cortex during reward anticipation and with reduced connectivity with regions associated with inhibition and risk aversion, including the right inferior frontal gyrus
Disentangling the autism−anxiety overlap: fMRI of reward processing in a community-based longitudinal study	Mikita et al.	Translational psychiatry, 2016	Reward anticipation (*N* = 1472) Negative feedback (*N* = 1601) Positive feedback (*N* = 1726)	Baseline (14.4, SD not reported) Feedback (~16, SD not reported)		Participants with autism spectrum disorder (ADS) traits had reduced BOLD responses in dorsal prefrontal regions during reward anticipation and negative feedback (*p* = 0.001). High anxiety symptoms were correlated with increased lateral prefrontal responses during reward anticipation and decreased responses to reward feedback (*p* < 0.05). Interaction between ASD and anxiety showed significantly lower activations compared to ASD alone.
The brain’s response to reward anticipation and depression in adolescence: dimensionality, specificity, and longitudinal predictions in a community-based sample	Stringaris et al.	The American Journal of Psychiatry, 2015	Healthy subjects Baseline (*N* = 123) Follow-up (*N* = 902) Subthreshold depression Baseline (*N* = 101) Follow-up (*N* = 68) Clinical depression Baseline (*N* = 22) Follow-up (*N* = 29)	Healthy subjects Baseline (14.4, SD 0.4) Follow-up (16.4, SD 0.4) Subthreshold depression Baseline (14.5, SD 0.4) Follow-up (16.4, SD 0.4) Clinical depression Baseline (14.4, SD 0.3) Follow-up (16.5, SD 0.3–0.5)		Bilaterally lower vs activation elicited by reward anticipation in groups with subthreshold and clinical depression compared to healthy group (*p* < 0.005). Low ventral left (EF = 0.050) and right (EF = 0.047) striatal activation during reward anticipation predicted transition to subthreshold or clinical depression in previously healthy adolescents at 2-year follow-up
No differences in ventral striatum responsivity between adolescents with a positive family history of alcoholism and controls	Müller et al.	Addiction Biology, 2014	Family history positive (FHD+) (*N* = 206) Family history negative (FHD−) (*N* = 206) FH+ (*N* = 77) FH− (*N* = 77)	FHD+ (14.7, SD 0.4) FHD− (14.7, SD 0.3)		Reward anticipation as well as reward feedback elicited activation in the ventral striatum in all participants, no significant differences between adolescents with versus without a positive family history for alcohol use disorders
Altered reward processing in adolescents with prenatal exposure to maternal cigarette smoking	Müller et al.	JAMA Psychiatry, 2013	Participants exposed to intrauterine maternal smoking (*N* = 177) Nonexposed (*N* = 177)	Exposed (14.7, SD 0.4) Nonexposed (14.6, SD 0.4)		In adolescents prenatally exposed to cigarette smoke,reward anticipation but not feedback elicited a weaker functional activation of the right (EF = 0.04) and left (EF=) ventral striatum during reward anticipation
A target sample of adolescents and reward processing: same neural and behavioral correlates engaged in common paradigms?	Nees et al.	Experimental Brain Research, 2012	All participants (*N* = 54)	All Participants (~14, SD not reported)		Magnitude sensitive functional activation in VS response during reward anticipation (*p* = 0.036) and magnitude independent activation in the anterior cingulate cortex during feedback
Risk taking and the adolescent reward system: a potential common link to substance abuse	Schneider et al.	American Journal of Psychiatry, 2012	All participants (*N* = 266)	All participants (14.5, SD 0.4)		With increasing risk-taking bias, the ventral striatum showed decreased activation bilaterally during reward anticipation (EF = 0.57 and EF = 0.52)
Maternal interpersonal affiliation is associated with adolescents’ brain structure and reward processing	Schneider et al.	Translational Psychiatry, 2012	All participants (*N* = 63)	All participants (14.24, SD 0.25)		Maternal affiliation was significantly associated with ventral striatal (EF = 0.89) and caudate activation (EF = 1.1942) during reward feedback in female participants only
Determinants of early alcohol use in healthy adolescents:the differential contribution of neuroimaging and psychological factors	Nees F et al.	Neuropsychopharmacology, 2012	All participants (*N* = 324)	All participants (~14, SD not reported)		Reward-associated behavior, personality, and brain responses all contributed to alcohol intake with personality explaining a higher proportion of the variance (explained variance 16%) than behavior (explained variance 0.6%) and brain responses (explained variance 0.4%).
Lower ventral striatal activation during reward anticipation in adolescent smokers	Peters et al	The American Journal of Psychiatry, 2011	All participants (*N* = 86)	All participants (~14, SD not reported)		Neural responses in the ventral striatum during reward anticipation were significantly lower in the smokers than in the comparison subjects (*p* < 0.001), and in the smokers this response was correlated with smoking frequency.
Distinct brain structure and behavior related to ADHD and conduct disorder traits	Bayard et al.	Molecular Psychiatry, 2018	All participants (*N* = 1093)	All participants (14.47, SD 0.39)	Stop Signal Task (SST)	ADHD score correlated with SSRT (*p* = 0.021), while CD score did not (*p* = 0.740). This mirrored structural findings on prefrontal and anterior cingulate region.
Separate neural systems for behavioral change and for emotional responses to failure during behavioral inhibition	Deng et al.	Human Brain Mapping, 2017	All participants (*N* = 1709)	All participants (~14, SD not reported)		Succesful inhibition was related to activation in the lateral orbitofrontal cortex, inferior frontal gyrus and the dorsolateral prefrontal cortex (DLPFC) (*p* < 0.05). Second, the anterior cingulate and anterior insula (AI) were activated more on failure trials (*p* < 0.05)
Neural and cognitive correlates of the common and specific variance across externalizing problems in young adolescence	Castellanos-Ryan et al.	The American Journal of Psychiatry, 2014	All participants (*N* = 1778)	Baseline (14.4, SD 0.35) Follow-up (~ 16, SD not reported)		Impulsivity at age 14 significantly predicted the general externalizing factor at age 16, sensation-seeking at age 14 predicted substance misuse at age 16, and go/no-go commission errors as well as lower BOLD response in bilateral frontal cortex during failed inhibition at age 14 predicted ADHD/conduct disorder at age 16
Functional neuroimaging predictors of self-reportedpsychotic symptoms in adolescents	Bourque et al.	The American Journal of Psychiatry, 2017	Baseline (*N* = 300) Follow-up (*N* = 1196)	Baseline: subjects with psychotic-like symptoms (14.4; SD 0.31) versus no symptoms (14.35; SD 0.38). Follow-up at age 16	Emotional faces SST MID	Youths reporting psychotic-like experiencesshowed increased hippocampus/amygdala activity during processing of neutral faces (EF = 0.987). When controlling for baseline psychotic-like experiences and cannabis use, hyperactivation of the hippocampus/amygdala was the most prominent regional difference at age 16 in participants with mood fluctuation and psychotic symptoms versus subjects without such symptoms.
Psychosocial stress and brain function in adolescent psychopathology.	Quinlan et al.	American Journal of Psychiatry, 2017	All participants (*N* = 1288)	All participants (14.4, SD 0.40)	Emotional faces task	Conduct or hyperactivity/inattention symptoms in combination with a higher number of stressful life events showed stronger right amygdala activation (EF = 0.1733)
Neural correlates of three types of negative life events during angry face processing in adolescents	Gollier-Briant et al.	Social Cognitive and Affective Neuroscience, 2016	Baseline (*N* = 685) Follow-up (*N* = 523)	Baseline (14, SD not reported) Follow-up (16, SD not reported)		Lifetime ‘distress’ positively correlated with orbitofrontal (*p* = 0.005) and temporal cortex activations (*p* = 0.007) during angry face processing.
Cannabis use in early adolescence: evidence of amygdala hypersensitivity to signals of threat	Spechler et al.	Developmental Cognitive Neuroscience, 2015	All participants (*N* = 140)	All participants (14 SD not reported)		Higher amygdala activation elicited by angry versus neutral faces in cannabis users only, potentially indicating hypersensitivity to stress
Hormonal contraceptives, menstrual cycle and brain response to faces	Mareckova et al.	Social cognitive and affective neuroscience, 2014	All participants (*N* = 110)	All participants (14.5, SD not reported)		Response in the left FFA elicited by emotional faces was higher in the group taking contraceptives versus freely cycling females and during mid-cycle versus menstruation (EF = 0.49)
Do you see what i see? Sex differences in the discrimination of facial emotions during adolescence	Lee et al.	Emotion, 2013	All participants (*N* = 1951)	All participants (14 SD not reported)		Female participants showed faster and more sensitive perception of facial emotions than boys. Both sexes overidentified happiness and anger
Creating probabilistic maps of the face network in the adolescent brain: a multicenter functional MRI study	Tahmasebi et al.	Human Brain Mapping, 2012	All participants (*N* = 1110)	All participants (14.5, SD 0.4)		Identification of 21 brain regions with high probability for responding to faces. Stronger neural response to ambiguous faces in the fusiform face area and further regions in female versus male adolescents, slightly stronger response to angry faces in the amygdala of male versus female adolescents
Boys do it the right way: sex-dependent amygdala lateralization during face processing in adolescents	Schneider et al.	Neuroimage, 2011	Female (*N* = 235) Male (*N* = 235)	Female(14.48, SD 0.4) Male (14.47, SD 0.4)		Emotional faces elicit stronger right amygdale activation in males versus females (EF = 0.3279)

Another 41 papers investigated methodological or statistical approaches or the associations of some other variables not listed above (e.g., maternal smoking and video gaming) on brain imaging parameters and behavioral variables and therefore were not included in the supplementary tables [12, 78–117].

**Fig. 2:**
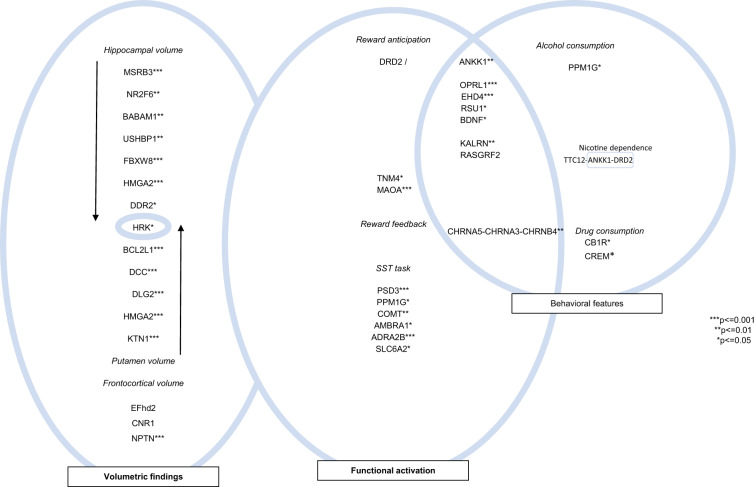
Effect of genetic variations on brain structure, function and behavior. Genes and epigenetic modifications associated with brain structure (hippocampus and putamen volume), functional activation (reward anticipation and feedback as elicited by the MID task; SST task) and behavior (alcohol consumption, nicotine and cannabis consumption).

From the multitude of findings, we here focus on brain regions relevant for drug use, including the hippocampus, striatum and frontal cortex. Drug use frequently starts during adolescence and has been a focus of IMAGEN research [55–59]. Figure 2 illustrates the potential of a simultaneous assessment of the effect of genetic variations on brain structure, function and behavior, using as an example the effects of genetic variation on (1) the volume of frontal, hippocampal, amygdala and striatal brain areas, (2) functional activation during reward anticipation and feedback, behavioral inhibition, and (3) alcohol, tobacco, and cannabis consumption. We also indicate whether structure or function of the respective brain regions were themselves directly associated with drug consumption.

### Genotype effects on brain structure relevant for drug use

With respect to brain structure, frontocortical, hippocampal, and striatal (especially putamen) volumes were associated with variation in genes that contribute to metabolic and endocrine function, cell cycle and apoptosis, neurodevelopment and neurotransmission (Table 1). Notably, only variation in the HRK (“Between Harakiri, BCL2 Interacting Protein”) gene, which has been associated with cell cycle and apoptosis [20], contributed to both hippocampus and putamen volume [4, 25] (Fig. 2), indicating that the genetic contribution to brain volumes in adolescence may be rather site-specific. The other polymorphisms associated with brain volume at age 14 contribute to metabolic and endocrine function (APOE), cell cycle and apoptosis (BABAM1, FBXW8, HMGA2, DDR2, HRK, BCL2L1, HMGA2, and Efhd2), neurodevelopment (NR2F6, USHBP1, DCC, and FAT3), and neurotransmission (DLG2, CHR1, CNR1, and NPTN). The longitudinal design of the IMAGEN study will help to assess whether the effects of these genetic variations on brain volume are also age dependent [4, 14, 19, 22–28].

### Genotype effects on functional brain activation relevant for drug use

With respect to reward anticipation and feedback (Table 2 and Fig. 2), functional activation elicited by the MID task was associated with polymorphisms in genes influencing neurodevelopment (TNM4 and BDNF) [15, 39] and neurotransmission (DRD2/ANKK1and MAOA) [36, 38]. Further, lower striatal activation during reward anticipation was associated with a high risk-taking bias (i.e., a strong tendency to engage in risky behavior) [59], while no significant difference was observed between adolescents with and without family history of alcohol use disorder [56].

With respect to the SST, distinct brain networks were associated with drug use versus attention deficit-hyperactivity symptoms; this study also reported that genetic variation in the norepinephrine transporter gene SLC6A2 was associated with the use of illegal substances but not functional activation elicited by behavioral inhibition [18]. The Arf6 activator Efa6/PSD3 was associated with ethanol-induced sedation and reduced tolerance development in drosophila and with altered prefrontal cortex activation during behavioral inhibition in the IMAGEN sample [46]. Moreover, epigenetic variation in the PPM1G gene locus was associated with increased functional activation of the right subthalamic nucleus during behavioral inhibition [16], and epigenetic modification of the OPRL1 gene mediated the effect of psychosocial stress and neutral striatal activation in the MID task on binge drinking in adolescence [31].

Regarding the EF paradigm, genetic variation in CB1R was associated with activation of the bilateral amygdala to angry faces, which in turn was correlated with higher drug intake [18].

### Genotype and other associations with drug use

With respect to alcohol consumption, Fig. 2 shows genes associated with alcohol intake in the 30 days before study inclusion. The EHD4 gene, which has been associated with the cell cycle and apoptosis [21], showed a statistically significant association with alcohol consumption at age 14 [34]. Neuroimaging data additionally showed that low functional activation of the ventral striatum and of the putamen during reward anticipation at age 14 predicted high alcohol intake at age 16, with the association between functional putamen activation and alcohol intake being mediated by the BDNF Val66Met polymorphism [19]. The above mentioned epigenetic variation in the PPM1G gene was associated with high impulsiveness and early escalation of alcohol use [16]. Also, the human ortholog sof the Arf6 and Erf6 genes were associated with increased frequency of drinking and binge drinking episodes [46].

With respect to cannabis and other illegal drugs consumption, genetic variation in the norepinephrine transporter gene was associated with hypoactivation of the right inferior orbitofrontal network and speed of motor inhibition during SST, which was correlated with higher drug intake [18].

With respect to nicotine consumption, lower striatal activation during reward anticipation correlated with prenatal exposure to maternal smoking [57]. Risk variant rs578776, a variant in the CHRNA5–CHRNA3–CHRNB4 gene cluster, influences susceptibility to nicotine dependence by dampening the response of the anterior cingulate cortex to reward feedback, without recruiting the striatum or orbitofrontal cortex during feedback or anticipation [37].

## Discussion

The IMAGEN study has identified several genetic polymorphisms that interact with adolescent brain function and behavior, thus helping to disentangle their general functional roles [16, 46, 50]. With 2000 adolescents aged 14 when included in the study, the IMAGEN cohort was the largest sample available to date for imaging genetics analyses, thus improving power compared with previous studies with smaller sample sizes [118]. Due to its longitudinal design, the IMAGEN study can help to reveal gene-environment interactions in the manifestation of mental disorders from adolescence to and young adulthood.

From the large body of IMAGEN publications, we focused on findings of genes interacting with (1) brain volume, (2) functional activation, and (3) drug intake, which tends to start during adolescence and has repeatedly been attributed to gene-environment interactions [18, 55]

To explore psychopathology-relevant brain functions, we selected tasks that probe key aspects of reinforcement-related behaviors and emotional processing implicated in frequent neuropsychiatric disorders: response inhibition, emotional reactivity, and reward sensitivity. The neuroimaging tasks were chosen because they reliably elicit strong activation in functional networks underlying inhibitory control (SST) [119], emotional reactivity to social stimuli (EF) [120] and reward anticipation/outcome (MID) [121]. Some limitations of using these three tasks include incomplete assessment of different aspects of behavioral constructs, e.g., SST measuring motor impulsivity but not delay discounting, which was assesed behaviorally; passive reception of socially salient stimuli (faces) outside of social context; lack of possibilities for computational modeling of reward-related decisions in the MID task.

The IMAGEN study has assessed a wide range of environmental factors including childhood trauma, bullying, stressful life events, family mental health, pre- and peri-natal events, and family conflict. For example, peer victimization was indirectly associated with increased anxiety via decrease in left putamen and caudate volume [114]. Moreover, stressful life events were associated with the interaction between functional amygdala activation elicited by the EF task in adolescents with conduct and hyperactivity symptoms [71]. We here describe genetic and environmental effects on brain structure, function, and behavior assessed in the IMAGEN study.

Regarding reward anticipation and feedback, the IMAGEN consortium focused on polymorphisms implicated in both impulsive and addictive behavior. Reduced ventral striatal activation during reward anticipation has repeatedly been observed in alcohol-dependent patients and may be attributed to dopamine dysfunction following detoxification [2, 122]. However, it was unclear whether reduced activation during reward anticipation is present prior to the development of alcohol use or alcohol use disorder. Here, Büchel et al. [55] observed that reduced functional activation of the ventral striatum, midbrain, and prefrontal cortex at age 14 predicts drug use at age 16. Environmental factors that may contribute to such a blunted ventral striatal activation include maternal smoking during pregnancy [57]. Blunted ventral striatal activation in adolescents has been associated with increased impulsivity [60], a finding that replicates a similar observation in adult alcohol-dependent patients and controls [123]. Moreover, impulsivity has been associated with early life stress, [2] and methylation of the OPRL1 gene was shown to mediate the effect of psychosocial stress on impulsive alcohol intake (binging) and the associated ventrostriatal activation in adolescence [31]. The OPRL1 genotype has repeatedly been replicated on alcohol use disorders [124, 125].

Beyond environmental factors, genetic variance contributes to reduced ventral striatal activation during reward anticipation elicited by cues that predict reward. In humans, Stacey et al. [40] observed an association between reduced functional activation of the ventral striatum and a haplotype block containing the Rasgrf2 polymorphism rs26907, which was associated with alcohol intake in a previous meta-analysis [126]. In animal experiments, Stacey et al. [40] observed that Rasgrf2 knockout mice displayed a significant reduction in ethanol intake relative to wild type controls, lower intake at the highest ethanol concentrations, and blunted diurnal drinking. Rasgrf2 knockout mice also showed a significant reduction in ethanol preference, which was measured by the percentage of ethanol relative to water intake [40]. In addition, Rasgrf knockout mice showed a diminished noradrenergic and serotonergic response in the ventral striatum/nucleus accumbens and a decrease in β1 adrenoreceptor gene expression [127]. Easton and colleagues have shown that Rasgrf2 mediates the presence of noradrenaline (NA) and serotonin (5-HT) in the synaptic cleft at both basal level and after acute alcohol exposure. After subchronic alcohol exposure, the NA system is modified, and Rasgrf2 regulates alterations in expression levels of adrenoceptor mRNA. Rasgrf2 may be a mechanism by which the NA system is prevented from adapting to subchronic alcohol intake, which could in turn influence vulnerability to the effects of repeated alcohol exposure [128]. Moreover, it was shown that Rasgrf2 controls dopaminergic signaling and adaptations to alcohol also in other brain regions, beyond the nucleus accumbens [127]. Another Rasgrf2 polymorphism (rs2369955) was associated with current and future binge drinking in the IMAGEN cohort [19]. Therefore, the IMAGEN findings regarding Rasgrf2 are neurobiologically plausible, however, replication in an independent imaging sample has yet to be done.

These IMAGEN findings can be explained by the fact that Rasgrf2 interacts with activation of the MAPK/ERK pathway, which is involved in neurotransmission through dopamine receptors and transporters and thus potentially associated with dopamine-dependent reward mechanisms in alcoholism [129, 130]. In mouse models, ethanol administration increased ERK activity in the nucleus accumbens, and inhibition of ERK activity influenced ethanol self-administration [19].

Further genetic variance impacting on ventral striatal activation was discovered by Nees et al. [15], who showed that BDNF Val homozygotes compared with Met carriers had lower putamen reactivity during reward anticipation. This is in partial accordance with results from Pecina et al., who also observed effects of BDNF ValMet genotype on functional activation during the anticipations of rewards and losses, albeit only significant during loss anticipation [131]. Lower striatal activation during reward anticipation may impact on personality traits reflecting the orientation towards positive reinforcers. Benzerouk et al. [132] indeed observed that BDNF Val carriers with a positive family history for alcohol use disorders displayed lower levels of reward dependence compared to probands without such a family history. ValMet genotype was also associated with risk of relapse in alcohol dependence, however, no functional brain imaging was performed in the study [133]. Furthermore, the BDNF Val68Met polymorphism regulates BDNF expression and was also implicated in rodent models of uncontrolled and excessive alcohol intake [134]. BDNF expression was lower in the nucleus accumbens [135] and in the central and medial nucleus of the amygdala of alcohol preferring versus non-preferring rats [136], and short versus long-term alcohol intake differentially regulate BDNF mRNA levels in the nucleus accumbens in rats actively versus passively consuming alcohol [137].

With respect to a dimensional approach towards mental disorders [138], the IMAGEN consortium also assessed whether blunted ventral striatal activation elicited by reward indicating cues is associated with psychotic or affective symptoms and disorders. Previous studies showed that low functional activation of the ventral striatum during reward anticipation in patients with depression and schizophrenia was related to the severity of negative mood states including depression and anhedonia [139, 140]. In the IMAGEN adolescents, Stringaris et al. [54] observed that low ventral striatum activation during reward anticipation at age 14 predicted transition to subthreshold or clinical depression in previously healthy adolescents at age 16. Vulser et al. [89] observed that adolescents with subthreshold depression also had smaller gray matter volumes in the ventromedial prefrontal and rostral anterior cingulate cortex as well as in the putamen [25]. In 2018, Vulser et al. showed that early fractional anisotropy variations in tracts projecting from the corpus callosum to the anterior cingulate cortex may denote a higher risk of transition to depression in adolescents [112]. Findings regarding the cingulate cortex volume are interesting in light of their association with major depression [141] and the role of this brain area in error detection and behavioral control [142], which may also play a role in recovery from affective disorders. Indeed, patients with sustained activation elicited by reward feedback across task runs in the anterior cingulate cortex were more responsive to behavioral activation therapy [143].

With respect to higher cognitive functions, Nymberg et al. [36] showed that higher ventral striatal and caudate activation during reward feedback was associated with higher working memory performance and that this interaction was limited to A allele carriers of the DRD2/ANKK1 polymorphism. Also Taq1A of the ANKK1 gene interacted with a lateral orbitofrontal activation during reward anticipation [45] and predicted alcohol drinking 2 years later [105]. These findings provide evidence for an interaction between reward processing and complex cognitive capacities [144, 145]. Differences in striatal dopaminergic neurotransmission have also been associated with differences in working memory performance [146, 147], in line with findings concerning dopaminergic markers and cognitive capacity [65].

Beyond reward anticipation and feedback, impulsivity as operationalized by behavioral inhibition has been implicated in the development and maintenance of substance use disorders [148]. The subthalamic nucleus modifies information processing in fronto-striatal networks relevant for impulse control [148, 149]. Increased functional activation of the subthalamic nucleus was associated with hypermethylation in the PPM1G gene, which also correlated with increased impulsiveness and alcohol use in adolescence [16]. Further brain areas implicated in behavioral inhibition are the prefrontal cortex [18], whose activation was associated with the human ortholog of Arf6 and orthologs of Efa6 (PSD1-4) [46].

Regarding the processing of affective faces, the study of Spechler et al. shows that stronger amygdala activation to signals of threat was associated with high cannabis intake. This observation points to another neurobiological dimension contributing both to negative mood states and drug intake [72]. Indeed, increased amygdala activation towards aversive stimuli has been shown to contribute to anxiety, feelings of being threatened and aggression, which in return predict excessive alcohol use [148]. Stronger amygdala activation during emotional processing was also observed in adolescents who experienced both a high number of stressful life events and strong symptoms of conduct disorder and hyperactivity [71], highlighting how experience can modulate brain-behavior relationships.

Overall, the IMAGEN consortium was able to detect a number of genetic polymorphisms with specific effects on brain structure and function and their association with symptoms of mental disorders in adolescents. IMAGEN’s study design has supported the elucidation of biological and environmental mechanisms of substance use-related behaviors, but we note limitations and ways to address them. The rather large-sample size overcomes some of the limitations of previous genetic imaging studies, however, independent replications are required. As studies like IMAGEN have their data repeatedly used by researchers, which—if systematic errors existed in the data would then be propagated into all research output, strict quality assurance and quality control measures are taken by the central database team that has overseen data management across all study time points. This allows for quick identification and resolution of errors that could occur before data are released for analyses (i.e., data entry and data upload/transfer).

The very nature of multivariate multimodal approaches bears a multiple comparison issue. Fortunately, clinical samples and other large phenotypically rich studies such as UK Biobank provide the opportunity to externally validate IMAGEN findings. Resampling methods such as bootstrapping and cross-validation (e.g., leave-one-out and split-half) are techniques we have used to internally validate model fit and estimate a model’s generalizability [41]. Moreover, the IMAGEN consortium asks for research proposals before data access is granted, and thus prevents unethical data dredging. Nonetheless, exploratory data analysis can be performed and has to be clearly described as such. Replication of such a cohort might be very costly, that is why rigorous statistics including the above mentioned internal cross-validation for estimation of predictive power and the reporting of confidence intervals in order to describe a range of plausible estimates should be promoted. With respect to intraindividual variance, reliability measures as well as developmental changes replications can be carried out within the IMAGEN sample, e.g., by assessing whether the same polymorphisms impact on subcortical brain volumes at age 14 and 16 or in young adulthood. Given the multisite design, variance from different acquisition sites were considered based on a central protocol.

The issue of participant attrition over time may limit some longitudinal multimodal statistical modeling and internal validation methods (i.e., due to reduced power and incomplete data), but with more than 1300 participants assessed at age 22, there is still great opportunity to understand the influence of biology and the environment on developmental trajectories of substance use. Relevant to identifying gene-environment interactions on brain and behavior, the relatively homogeneous socioeconomic background of the Western European participating families meant IMAGEN was not well placed to investigate particular environmental influences such as nutrition or hazardous exposures. Furthermore, no resources were available to independently confirm the reliability of the self-reported environmental data.

For the purpose of genetic analyses, the IMAGEN participants are of European ancestry—as a result the generalizability of findings to other cultures and stress/environmental exposures is unknown. Therefore, future replications should also be carried out outside of Europe, requiring careful harmonization of clinical tools and paradigms. As recently reported [150], the IMAGEN study has aligned itself with other global neuroimaging-genetics adolescent cohorts that will further enhance and clarify the work presented here.
